# Fabrication of Cellulose Nanocrystal-g-Poly(Acrylic Acid-Co-Acrylamide) Aerogels for Efficient Pb(II) Removal

**DOI:** 10.3390/polym12020333

**Published:** 2020-02-05

**Authors:** Yifan Chen, Qian Li, Yujie Li, Qijun Zhang, Jingda Huang, Qiang Wu, Siqun Wang

**Affiliations:** 1 School of Engineering, Zhejiang A & F University, Hangzhou 311300, China; yfchen.28425@foxmail.com (Y.C.); liqian_polymer@126.com (Q.L.); liyujie19971006@163.com (Y.L.); hjd1015@163.com (J.H.); 2 Center for Renewable Carbon, University of Tennessee, Knoxville, TN 37996, USA; qzhang37@vols.utk.edu; 3 Zhejiang Provincial Collaborative Innovation Center for Bamboo Resources and High-Efficiency Utilization, Hangzhou 311300, China

**Keywords:** cellulose nanocrystal, graft copolymerization, aerogel, adsorption, lead

## Abstract

In this work, cellulose nanocrystals (CNCs) obtained by the acid hydrolysis of waste bamboo powder were used to synthesize cellulose nanocrystal-g-poly(acrylic acid-co-acrylamide) (CNC-g-P(AA/AM)) aerogels via graft copolymerization followed by freeze-drying. The structure and morphology of the resulting aerogels were characterized by Fourier transform infrared spectroscopy (FTIR) and scanning electron microscopy (SEM), and the CNC-g-P(AA/AM) aerogels exhibited excellent absorbent properties and adsorption capacities. Subsequent Pb(II) adsorption studies showed that the kinetic data followed the pseudo-second-order equation, while the adsorption isotherms were best described using the Langmuir model. The maximum Pb(II) adsorption capacity calculated by the Langmuir model reached up to 366.3 mg/g, which is a capacity that outperformed that of the pure CNC aerogel. The CNC-g-P (AA/AM) aerogels become structurally stable through chemical cross-linking, which enabled them to be easily regenerated in HCl solution and retain the adsorption capacity after repeated use. The aerogels were found to maintain 81.3% removal efficiency after five consecutive adsorption–desorption cycles. Therefore, this study demonstrated an effective method for the fabrication of an aerogel adsorbent with an excellent reusability in the effective removal of Pb(II) from aqueous solutions.

## 1. Introduction

Over the past few decades, contamination from lead has caused major public health issues due to the widespread usage of this element in many important industries, such as battery manufacturing, electroplating, pigments, and finishing [1,2]. Given the non-biodegradability and cumulative toxicity of lead, this contaminant slowly accumulates in human bodies through water intake or the food chain, ultimately resulting in serious diseases or even death [3,4]. To date, various methods have been applied for the treatment of lead-containing wastewater, including chemical precipitation [5], ion exchange [6], membrane filtration [7], electrochemical technologies [8], and adsorption-based techniques [9,10]. Among them, adsorption is considered to be superior to other technologies due to its ease of operation, high selectivity, high removal efficiency at low concentrations, and lack of secondary pollution [11,12,13,14]. In addition, since the quality of the treated water is directly dependent on the properties of the adsorbent, it is of great importance to develop adsorbents that exhibit excellent performances.

Activated carbon is currently the most widely used adsorbent in the area of heavy metal decontamination. However, its low selectivity and regeneration problems restrict its use [15]. Indeed, activated carbon is not well suited for the treatment of industrial effluents containing low concentrations of Pb(II), and as such, the development of new high-efficiency adsorbents as an alternative to activated carbon is necessary [16]. Since such adsorbents should be based on readily available and low-cost raw materials, cellulose, the most abundant biopolymer, has attracted increasing attention as a promising adsorbent material due to its renewability and eco-friendly nature.

In recent years, special attention has also been given to nanocellulose. As a cellulose derivative with a large specific surface area, high functionality, light weight, mechanical strength, and tunable surface chemistry, nanocellulose exhibits great potential for application in wastewater decontamination [17,18]. Although nanocellulose has a limited adsorption capacity, its structure can be modified or grafted to introduce specific functional groups (e.g., −COOH, −NH_2_, or −SO_3_H) that can further enhance its binding efficiency toward pollutants [19,20,21]. For example, cellulose nanocrystals (CNCs) prepared by acid hydrolysis exhibit a greatly enhanced adsorption capacity following succinic acid modification, giving a maximum adsorption capacity for Cd(II) of 259.7 mg/g [22]. In addition, COO^−^-modified cellulose nanofibers (CNFs) presented a significant enhancement in the adsorption capacities toward Ni(II) and Cr(III) as well as Cd(II) and Pb(II) compared to unmodified CNFs. Furthermore, thiol-modified nanofibrillated cellulose (NFC) was able to effectively remove Hg(II) ions over a wide range of concentrations and pH values [23].

Although modified nanocellulose exhibits an excellent adsorption capacity, some limitations remain. For example, recovery of the nanocellulose is challenging after its use in water treatment processes due to its tendency to agglomerate and also due to its nano-sized dimensions, thereby rendering it unsuitable for direct use as an adsorbent [24]. To address this issue, nanocellulose is often converted into an aerogel or film by appropriate cross-linking [25]. However, the majority of aerogels are prepared using physical interactions, and so the resulting poor chemical stability and low strength in practical applications can also present issues in their application [26].

Thus, to enhance the binding ability of nanocellulose to metal ions, the grafting of commonly used vinyl monomers such as acrylic acid and acrylamide onto the nanocellulose surface results in the introduction of metal-binding carboxyl and amide groups. In addition, a certain degree of wet strength can be obtained via the appropriate cross-linking to achieve desirable separation properties. It should also be noted that since nanocellulose regeneration has received little attention to date, additional studies are required to evaluate the reusability of nanocellulose-based adsorbents.

Thus, we herein report the preparation of CNC-based aerogels exhibiting high adsorption capacities and excellent reusability properties, in which poly(acrylic acid-co-acrylamide) (P(AA/AM)) is grafted onto CNC to increase its adsorption capacity, and *N*,*N*’-methylenebisacrylamide (MBA) is used to cross-link the cellulose nanocrystal-g-poly(acrylic acid-co-acrylamide) (CNC-g-P(AA/AM)) and enhance the wet strength. The CNC used in this study is prepared by sulfuric acid hydrolysis from industrial bamboo waste powder, which was found to have a high surface charge (−38.7 mV), a size of 174 ± 60 nm in length and 8.5 ± 2.5 nm in diameter, and a sulfonate content (0.33 mmol/g). The prepared aerogels are then employed in the removal of Pb(II) from aqueous solution. Furthermore, the data obtained from the adsorption experiments are assessed by various kinetic and isotherm models to study the adsorption mechanism. Finally, the reusability of each CNC-g-P(AA/AM) aerogel is also investigated as an important performance parameter.

## 2. Experimental Section

### 2.1. Materials

Bamboo powder (the CNC raw material) was collected from Hangzhou Bamfox Bamboo Products Co., Ltd. (Hangzhou, China). Sulfuric acid (H_2_SO_4_, 98 wt %) was purchased from Xilong Chemical Co., Ltd. (Yichang, China). Acrylic acid (AA), sodium hypochlorite (NaClO_2_), and sodium hydroxide (NaOH) were purchased from Aladdin Chemistry Co., Ltd. (Shanghai, China). Toluene, ethanol, and acetic acid were supplied by Sinopharm Chemical Reagent Company (Shanghai, China). Acrylamide (AM), *N*,*N*’-methylenebisacrylamide (MBA), and potassium persulfate (KPS) were provided by Macklin Biochemical Co., Ltd. (Shanghai, China). All reagents were of analytical grade and were used without any further purification. All aqueous solutions and suspensions were prepared using deionized water.

### 2.2. Preparation of the CNCs

The CNCs were prepared from waste bamboo power according to a previously reported pretreatment and sulfuric acid hydrolysis method [27]. More specifically, the bamboo powder (30 g) was immersed in a toluene-ethanol solution (450 mL, 2:1 v/v) for 20 h under magnetic stirring and then washed with ethanol to remove any wax. Subsequently, the reaction mixture was transferred into a beaker containing a 1.4 wt % NaClO_2_ solution (1000 mL, pH 3–4, adjusted with acetic acid), and stirred in a water bath at 70 °C for 5 h to remove lignin. After this time, the obtained powder was immersed in a 5 wt % NaOH solution (600 mL) and stirred at 90 °C for 4 h to remove the hemicellulose and obtain the bamboo cellulose. For preparation of the CNC suspensions, the bamboo cellulose was hydrolyzed using a 65 wt % sulfuric acid solution (10 mL g^−1^) at 45 °C under constant mechanical stirring, and the reaction was stopped by diluting with 10 times the volume of ice water. The resulting suspension was subjected to centrifugation, and then dialyzed until a neutral pH was achieved. Finally, the suspension was subjected to ultrasonication using an ultrasonic processor (JY98-IIID Ningbo Scientz Biotechnology Co., Ltd., Ningbo, China) in an ice bath to obtain a uniformly dispersed CNC suspension.

### 2.3. Synthesis of the CNC-g-P(AA/AM) Aerogels

**Figure 1:**
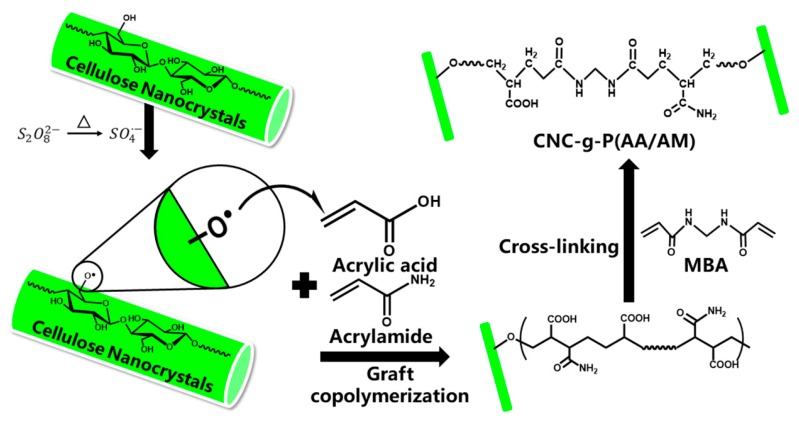
Mechanism of cellulose nanocrystal-g-poly(acrylic acid-co-acrylamide) (CNC-g-P(AA/AM)) aerogel preparation.

The CNC-g-P(AA/AM) aerogels were synthesized by grafting the CNCs with AA and AM and cross-linking with MBA, as depicted in Figure 1. Initially, different weights of the CNC slurry (3.0, 6.0, or 12.0 g by dry weight) were added to water (total 150 g suspension) under continuous stirring followed by ultrasonication for 10 min. Then, each resulting suspension was added to a separate three-necked flask equipped with an electric stirrer, and nitrogen gas was bubbled through the suspensions for 30 min to remove any dissolved oxygen. After this time, KPS (0.04 g) dissolved in deionized water (5 g) was added to each suspension, and the resulting mixtures were maintained at 60 °C for 10 min. AA (3 g), AM (3 g), and MBA (0.08 g) dissolved in water (50g) were added to the above flasks and maintained under the same condition for a further 120 min to the obtain gel samples. Then, the resulting gels were neutralized by titration with sodium hydroxide solution washed with deionized water to remove any residual impurities. Finally, the samples were freeze-dried to obtain the desired aerogels, which were named based on the mass ratio of CNC to the total of AA and AM, namely CNC-g-P(AA/AM)-0.5, CNC-g-P(AA/AM)-1, and CNC-g-P(AA/AM)-2. P(AA/AM) without the addition of CNC was prepared in the same manner as a control group to investigate the effect of the addition of CNC on the swelling behavior of the aerogel. For a comparison of the adsorption performance, CNC was prepared into 2 wt % suspension and then freeze-dried to obtain CNC aerogel.

### 2.4. Characterization

#### 2.4.1. Fourier Transform Infrared (FTIR) Spectroscopy

Fourier transform infrared (FTIR) spectroscopy (Nicolet 6700, Thermofisher, Waltham, MA, USA) was used to identify the functional groups present on the CNC and the aerogels. The thoroughly dried aerogels were analyzed following careful grinding with KBr (1:200, w/w) and pressing into transparent pellets. Spectra were collected 32 times in the range 4000–500 cm^−1^ with a wavenumber resolution of 4 cm^−1^.

#### 2.4.2. Scanning Electron Microscopy (SEM)

The aerogel samples were observed by SEM, (TM3030, Tokyo, Japan) at an accelerating voltage of 15 kV. Prior to carrying out any observations, the aerogel surfaces were sputter-coated with a thin film of gold by an ion sputter (MC1000, Hitachi, Tokyo, Japan).

#### 2.4.3. Swelling Capacity and Kinetics

The dried sample (0.2 g) was fully immersed in excessive deionized water at 293 K until the swelling equilibrium was reached. At predetermined time intervals, samples were removed from the water and weighed after removing any water on the sample surface by wiping with wet filter paper. The swelling ratio (SR) was calculated according to Equation (1):

(1) $$\mathrm{SR}=\left(W_{S}-W_{d}\right)/W_{d}$$

where $W_{s}$ is the weight of the swollen aerogel and $W_{d}$ is the weight of the dry aerogel.

#### 2.4.4. Adsorption Studies

Adsorption experiments were conducted using the batch method to examine the adsorption kinetics and adsorption isotherms for the adsorption of Pb(II) by the CNC-g-P(AA/AM) aerogels. The adsorption kinetics were tested at 293 K by adding the dried sample (0.10 g) into the Pb(II) solution (100 mL) with a shaking rate of 150 rpm, where an initial Pb(II) concentration of 200 ppm was employed to reach the adsorption equilibrium. The variation in the concentration of Pb(II) was measured by an inductively coupled plasma optical emission spectrometer (NexION 1000, PerkinElmer, Fremont, CA, USA). The pseudo-first [28] and pseudo-second-order kinetic models [29] as well as the intraparticle diffusion model were used to determine the rate constant and to analyze the mechanism of the adsorption process:

(2) $$\ln\left(q_{e}-q_{t}\right)=\ln q_{e}-k_{1}t/2.303$$

(3) $$\frac{t}{q_{t}}=\frac{1}{k_{2}q_{e}^{2}}+\frac{t}{q_{e}}$$

where $q_{t}$ (mg g^−1^) and $q_{e}$ (mg g^−1^) are the amount of metal adsorbed at time *t* and at equilibrium, respectively, and $k_{1}$ (min^−1^) and $k_{2}$ (g mg^−1^·min^−1^) are the rate constants of the pseudo-first and pseudo-second order models, respectively.

Furthermore, the intra-particle diffusion model was also used to determine the mechanism of CNC-g-P(AA/AM) aerogels for Pb(II) removal in view of diffusion.

(4) $$q_{t}=k_{i}t^{1/2}+C$$

where $k_{i}$ is the intra-particle diffusion rate constant (mg g^−1^ min^1/2^) and C is a constant related to the boundary layer effect.

The Langmuir and Freundlich isotherm models are the most widely used sorption isotherms for examining the removal of metal ions from an aqueous solution. Therefore, they were employed here to evaluate the adsorption mechanism and to investigate the adsorption capacities of the adsorbents. The linear forms of the isotherm models are represented by Equations (5) and (6) [30,31]:

(5) $$\frac{C_{e}}{Q_{e}}=\frac{C_{e}}{Q_{m}}+\frac{1}{Q_{m}b}$$

(6) $$\mathrm{lg}Q_{e}=\mathrm{lg}k_{f}+\mathrm{lg}C_{e}/n$$

where $Q_{e}$ (mg g^−1^) is the equilibrium adsorption capacity, $C_{e}$ (mg L^−1^) is the equilibrium concentration of Pb(II), $Q_{m}$ (mg g^−1^) is the maximum adsorption capacity, b (L mg^−1^) is the Langmuir adsorption constant related to the energy of adsorption, and $k_{f}$ and n are the Freundlich adsorption constants that indicate the capacity and intensity of the adsorption, respectively.

## 3. Results and Discussion

### 3.1. Syntheses and Characterization of the CNC-g-P(AA/AM) Aerogels

The CNC-g-P(AA/AM) aerogels were prepared by free radical graft polymerization. During this process, the KPS initiator was firstly decomposed into sulfate anion radicals under heating. Then, the hydrogen atoms from the hydroxyl groups were trapped by these radicals to form alkoxy radicals on the CNC surface. Due to the functional group activity and steric effects, the primary hydroxyl groups present on CNC are more prone to attack by sulfate anion radicals than secondary hydroxyl groups [32]. With the introduction of monomer molecules (AA and AM) into the system, the active radical sites on CNC would initiate chain propagation in the monomer vinyl groups, thereby resulting in cross-linking reactions taking place simultaneously, and the MBA becoming connected to the polymer chains by covalent bonds to form a three-dimensional network. Finally, the samples were freeze-dried to obtain the desired CNC-g-P(AA/AM) aerogels. It should be noted here that chemical cross-linking is known to provide a certain degree of wet strength to aerogels, thereby preventing their collapse in water. According to the mass-volume method, the density of the resulting aerogels was measured as 11.2, 139.6, 85.3, and 55.2 mg/cm^3^ for CNC aerogel, CNC-g-P(AA-AM)-0.5, CNC-g-P(AA-AM)-1, and CNC-g-P(AA-AM)-2, respectively. To determine the structures of the prepared aerogels, characterization by FTIR and SEM was carried out.

**Figure 2:**
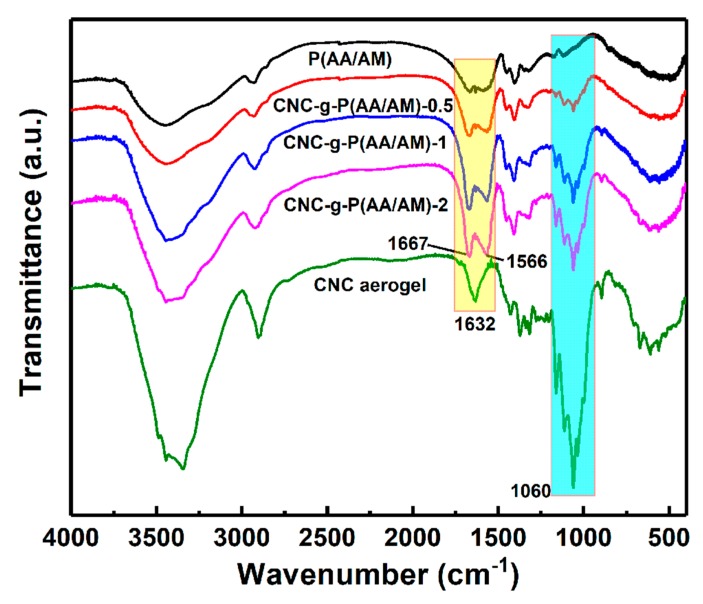
Fourier transform infrared spectroscopy (FTIR) spectra of the poly(acrylic acid-co-acrylamide) (P(AA/AM)), CNC-g-P(AA/AM)-0.5, CNC-g-P(AA/AM)-1, CNC-g-P(AA/AM)-2, and CNC aerogel.

To confirm the successful grafting of the P(AA/AM) chains onto the CNC surface, the functional groups of dry P(AA/AM), the CNC-g-P(AA/AM) aerogels, and the CNC aerogel were examined by FTIR, as outlined in Figure 2. The characteristic peaks observed in the CNC spectra at 3345 and 2904 cm^−1^ correspond to the O–H stretching and asymmetric C–H stretching, respectively. Meanwhile, the peaks at 1160 and 1060 cm^−1^ represent C–O and C–O–C stretching [33]; these signals are not observed in the spectra of P(AA/AM); therefore, their intensities reflect the CNC content. Furthermore, the C=O symmetric stretching vibration was observed at 1667 and 1451 cm^−1^ in the CNC-g-P(AA/AM) aerogel spectra, thereby confirming that AA and AM were successfully grafted onto the CNC surface [34,35]. Moreover, the bonding and bending vibration of N–H observed at 1566 cm^−1^ was attributed to the successful graft polymerization of the AM monomer. Shifting of the hydroxyl group peak from 3345 to 3445 cm^−1^ was attributed to the overlapping and intermolecular association of the O–H and N–H groups [36]. The signal peaks of O–H of carboxylic acid over 1700 cm^−1^ are not found in the FTIR spectra of CNC-g-P(AA/AM) aerogels due to the neutralization, but that of the carbonyl group of the carboxylate can be found at 1400 cm^−1^. Compared with the raw CNC, the peak corresponding to the C–H stretching vibration shifted from 2904 to 2928 cm^−1^ in the spectra of the CNC-g-P(AA/AM) aerogels, and it was interesting to note that the intensity of the peak attributed to the C–O and C–O–C stretching groups at 1060 cm^−1^ based on the normalized spectra was found to decrease upon decreasing the CNC content. This further confirmed that the P(AA/AM) was successfully grafted onto the CNC surface.

**Figure 3:**
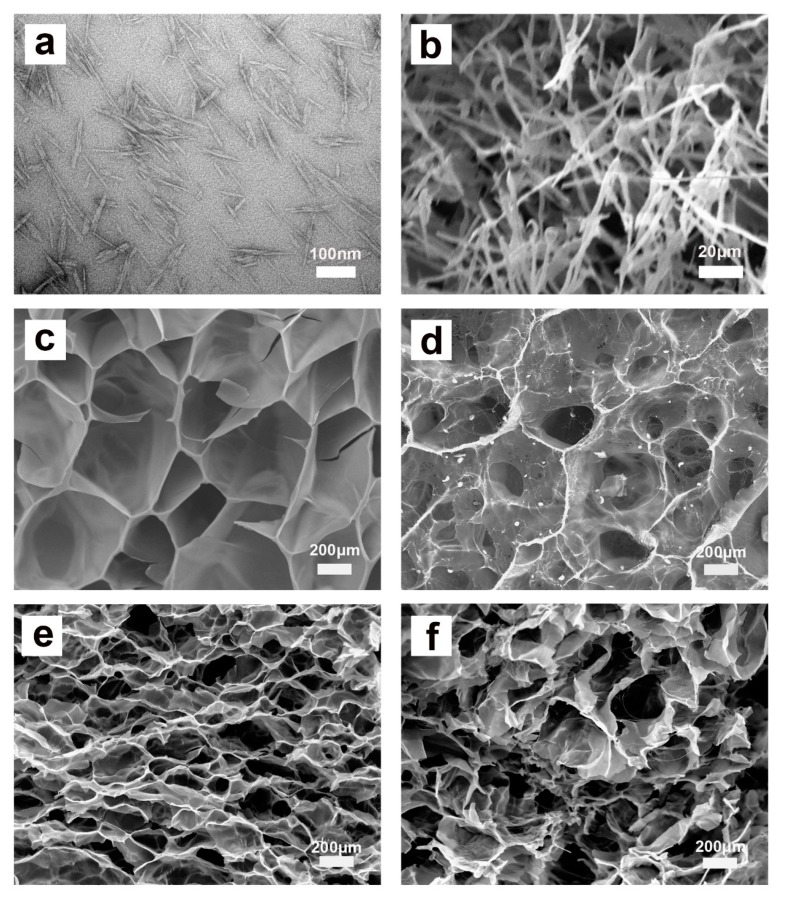
TEM image of CNCs (**a**) and SEM images of CNC aerogel (**b**), P(AA/AM) (**c**), CNC-g-P(AA/AM)-0.5 (**d**), CNC-g-P(AA/AM)-1 (**e**), and CNC-g-P(AA/AM)-2 (**f**).

The microstructure of the adsorbent is known to have a major impact on its adsorption performance. The TEM image of CNCs and SEM images of CNC aerogel, P(AA/AM), and CNC-g-P(AA/AM) aerogels are shown in Figure 3. As shown in Figure 3a, CNCs extracted from waste bamboo powder have good dispersibility, and needle-shaped fibers can be observed in the SEM image of CNC aerogel (Figure 3b). As for the chemical cross-linked aerogel, P(AA/AM) possesses an open and macroporous honeycomb structure, and comparison of the recorded SEM images shows that the pores of the aerogel become smaller upon increasing the CNC loading. This is because the addition of CNCs increased the effective cross-link density of aerogels and then led to an increase in the porosity of the aerogel, which may contribute to an increased specific surface.

### 3.2. Swelling Behavior

**Figure 4:**
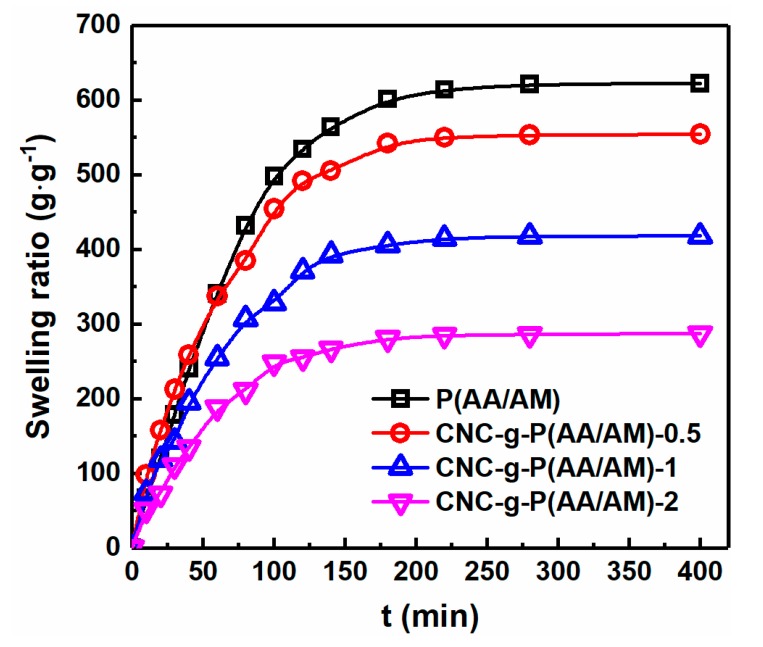
Swelling ratios of P(AA/AM), CNC-g-P(AA/AM)-0.5, CNC-g-P(AA/AM)-1, and CNC-g-P(AA/AM)-2.

The water absorbency capacity is an important parameter for evaluating gel materials since it is key to their application [37]. In the context of this study, CNC-g-P (AA/AM) aerogels have shown their ability to swell in water by absorbing water into their porous three-dimensional networks, while CNC aerogel does not. Therefore, the swelling properties of P(AA/AM), CNC-g-P(AA/AM)-0.5, CNC-g-P(AA/AM)-1, and CNC-g-P(AA/AM)-2 were examined and the results are presented in Figure 4. As shown, all aerogels exhibited a rapid water absorption behavior following immersion in water, and a time of approximately 3 h was required to attain the swelling equilibrium. Among the various aerogels examined herein, the swelling rate and swelling ratio of the P(AA/AM) aerogel was significantly greater than that of the CNC-g-P(AA/AM) aerogels. In addition, the maximum water absorption ratio of P(AA/AM) reached 621 c, while that of the other three CNC-g-P(AA/AM) aerogels was 553, 417, and 286 g g^−1^, respectively. This is because the addition of CNC leads to a shrinkage of the aerogel pores, which slows the entry of water, and it is also consistent with the results observed by SEM.

### 3.3. Adsorption Performance

**Figure 5:**
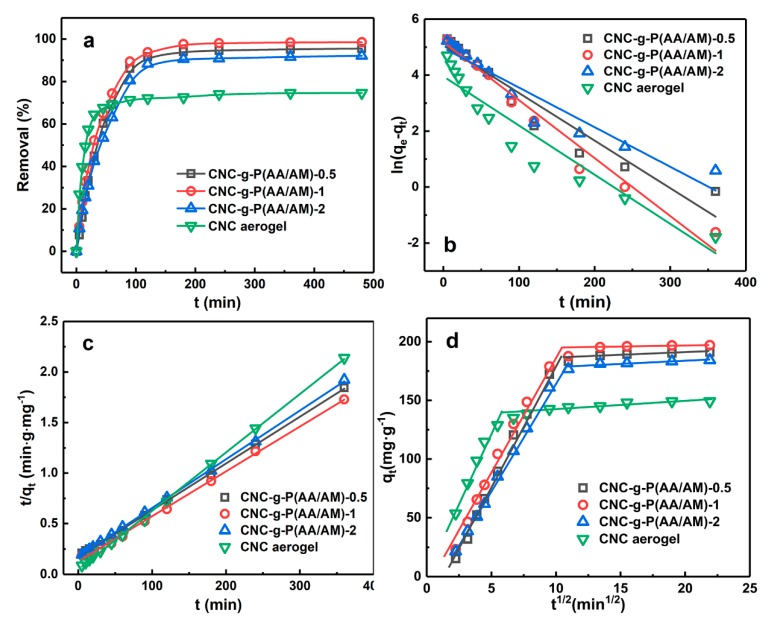
Kinetic studies of Pb(II) adsorption by the CNC-g-P(AA/AM)-0.5, CNC-g-P(AA/AM)-1, CNC-g-P(AA/AM)-2, and CNC aerogels (C_0_ = 200 mg L^−1^). (**a**) Effect of contact time, (**b**) the pseudo-first-order, (**c**) the pseudo-second-order, and (**d**) intra-particle diffusion models.

Then, the adsorption kinetics of the CNC-g-P(AA/AM) aerogels were investigated to determine the adsorption rate and eventually explore the adsorption mechanism involved. Since most heavy metals are often present in the form of divalent cations, and lead is the most common one, it is selected as the adsorption target. Thus, Figure 5a shows the variation in adsorption performance with respect to the contact time at an initial Pb(II) concentration of 200 ppm. As indicated, the CNC aerogel exhibited a high adsorption rate at the initial adsorption stage and reached the adsorption equilibrium rapidly with a final removal efficiency of 74.6%. In contrast, although the removal rates of the CNC-g-P(AA/AM) aerogels also increased rapidly during the initial stage, further increases were relatively slow, and equilibrium was finally reached after approximately 120 min. Impressively, the removal efficiencies of all the CNC-g-P(AA/AM) aerogels were >90%. Due to the porous structures of the aerogels and the presence of hydroxyl and sulfonic acid groups, Pb(II) can be rapidly absorbed by the aerogels in the initial stage. In addition, the presence of carboxylic acid and amino moieties led to a greater quantity of Pb(II) being adsorbed by the CNC-g-P(AA/AM) aerogels, thereby yielding significantly improved removal efficiencies compared to CNC itself.

#### 3.3.1. Adsorption Kinetics

To further analyze the adsorption process, the pseudo-first-order and pseudo-second-order kinetic models were used to investigate the adsorption kinetics (Figure 5b,c). The adsorption data in Figure 5a were fitted linearly using Equations (2) and (3), and the related kinetic parameters are summarized in Table 1. Among them, the coefficient of determination (R^2^) of kinetic models was used to analyze the fitting degrees with experimental data [38]. Thereby, the pseudo-second-order model better describes the adsorption kinetics of Pb(II) on the aerogels than the pseudo-first-order model [39]. In addition, the theoretical q_e2,cal_ calculated from the pseudo-second-order model agreed well with the experimentally-obtained equilibrium Pb(II) absorption (q_e,exp_), as outlined in Table 2. This also suggests that the adsorption reaction of Pb(II) was a chemical process controlled by chemisorption behavior [40].

The intra-particle diffusion model further reveals the adsorption mechanism of aerogel on Pb(II) from the perspective of ion diffusion. As shown in Figure 5d and Table 2, all the plots did not pass through the origin (0, 0) and include two linear segments, which indicated that the adsorption might be controlled by more than one adsorption mechanism. The first stage is the transport of Pb(II) from the solution to the aerogel external surface, and the second stage is when Pb(II) enters the interior of the aerogel by diffusion and combines with the active sites on the adsorbent [41]. Moreover, at the first stage, k_i_ of CNC aerogel was the highest, which suggested that Pb(II) are more likely to enter its interior. Since CNC-g-P(AA/AM) aerogels have 3D cellular structures, Pb(II) has to enter the interior of them through pores, which led to the CNC aerogel having a faster adsorption rate than them.

#### 3.3.2. Adsorption Isotherms

**Figure 6:**
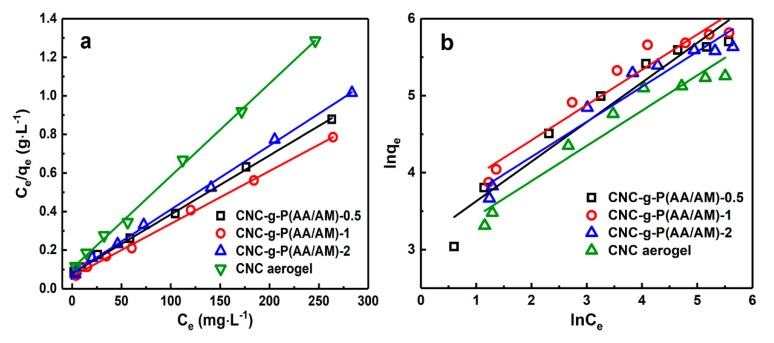
Comparison of the adsorption isotherm models for Pb(II) adsorption on the CNC-g-P(AA/AM)-0.5, CNC-g-P(AA/AM)-1, CNC-g-P(AA/AM)-2, and CNC aerogels. The (**a**) Langmuir and (**b**) Freundlich models.

To further explain the adsorption mechanism, the Langmuir and Freundlich isotherm models were employed; the adsorption isotherm parameters and corresponding plots are displayed in Figure 6 and Table 3, respectively. The higher correlation coefficient obtained for the Langmuir model illustrated that the experimental data fit the Langmuir model (R^2^ > 0.99) to a greater extent than the Freundlich model [42]. Based on these results, it can be deduced that the adsorption process involves Langmuir monolayer adsorption, which was likely due to electrostatic attractions between the carboxyl groups and Pb(II), in addition to chelation between the amine groups and the metal ions [43]. Among the three aerogels, CNC-g-P(AA/AM)-1 exhibited the highest adsorption capacity with a theoretical maximum adsorption capacity of 366.3 mg g^−1^.

### 3.4. Effect of Solution pH

**Figure 7:**
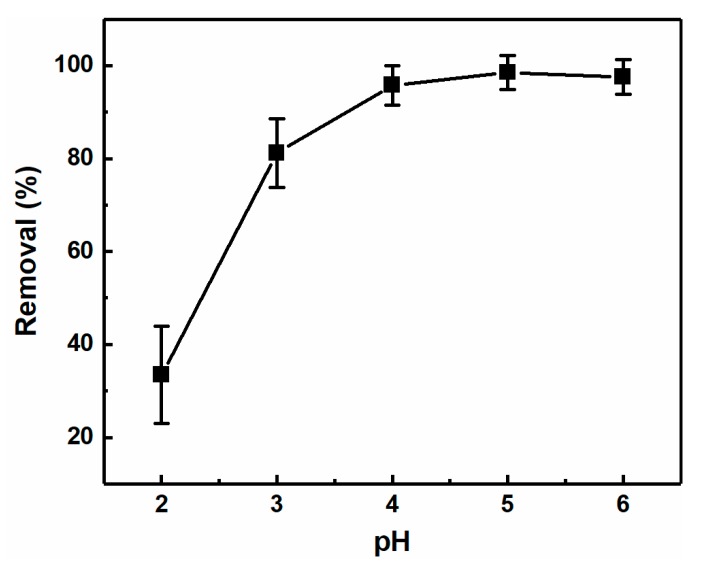
Effect of pH on the Pb(II) removal efficiency of CNC-g-P(AA-AM)-1 (C_0_ = 200 mg L^−1^).

The pH of the solution has also been considered to have an important influence on the metal ion adsorption process. Thus, using the CNC-g-P(AA/AM)-1 aerogel, the effect of the pH value (i.e., pH = 2–6) on its removal of Pb(II) was investigated. As outlined in Figure 7, an extremely low removal rate was obtained at pH = 2.0. Upon increasing the pH value, the removal efficiency increased significantly due to swelling of the aerogel and increased interactions between the aerogel and Pb(II). This can be accounted for by the fact that the amine and carboxyl groups are prone to protonation at lower pH values [44], thereby resulting in reduced electrostatic attraction to the positively charged Pb(II) ions. In addition, an excess of hydrogen ions reduces carboxyl ionization, leading to competition for the adsorption site. Therefore, the optimum pH range for Pb(II) removal was determined to be between pH = 4–6.

### 3.5. Reusability

**Figure 8:**
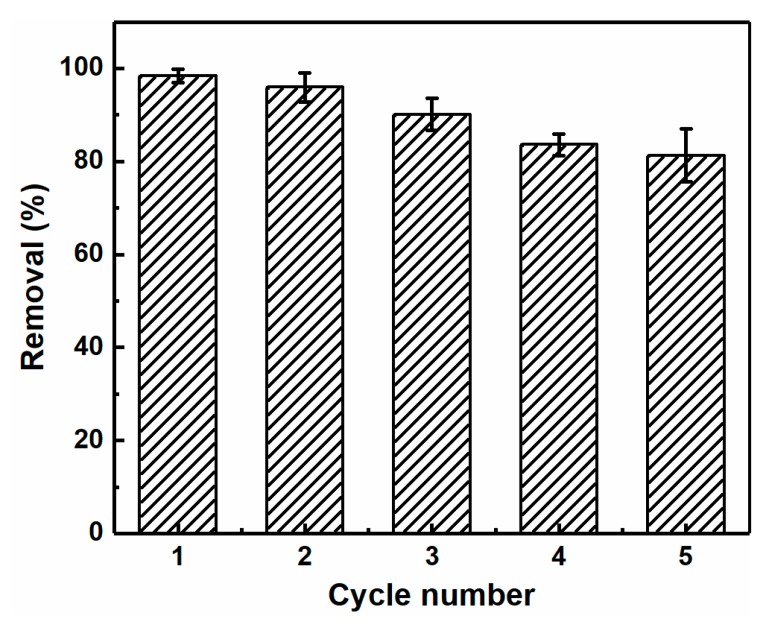
The removal efficiency of Pb(II) by CNC-g-P(AA/AM)-1 over five adsorption–desorption cycles.

Reusability is also an important factor for evaluating the performance of adsorbents in practical application. Thus, to regenerate the aerogels following adsorption, they were immersed in a 0.1 mol L^−1^ HCl solution, washed with deionized water, and employed in the subsequent adsorption cycles. As shown in Figure 8, after five adsorption–desorption cycles, the adsorption capacity of the CNC-g-P(AA/AM)-1 aerogel decreased slightly but remained at 81.3%. This demonstrated that the CNC-g-P(AA/AM)-1 aerogels exhibit excellent reusability properties. It is likely that the decrease in adsorption capacity was caused by the irreversible binding of Pb(II) to the active sites and protonation of the functional groups during the regeneration process.

Nowadays, nanocellulose-based adsorbents are of great interest. However, nanocellulose must be modified to achieve excellent adsorption capacity. The aerogels prepared in this work have shown better adsorption capacity than the nanocellulose-based adsorbents in many literatures [45,46,47]. Considering the excellent recyclability of CNC-g-P(AA/AM) aerogels, they are more competitive for practical use than many other nanocellulose-based adsorbents.

## 4. Conclusions

In this article, the CNCs extracted from waste bamboo powder using acid hydrolysis were demonstrated to be an effective adsorbent for removal of Pb(II) from aqueous solution and used to synthesize CNC-g-P(AA/AM) aerogels. The resulting aerogels exhibited excellent adsorption capabilities. Indeed, a theoretical maximum adsorption capacity of CNC-g-P(AA/AM) aerogels toward Pb(II) reached 366.30 mg/g, and this value is significantly higher than that of CNC alone, which is likely due to the structural modification and functionalization carried out herein. In addition, the pseudo-second-order and Langmuir models were found to best describe the kinetics and equilibrium data, respectively. The effects of CNCs dosage on the adsorption properties of CNC-g-P(AA/AM) aerogels were also systematically studied. Furthermore, CNC-g-P(AA/AM) aerogels could be regenerated by immersion in 0.1 mol L^−1^ HCl solution, and an 81.3% removal efficiency was maintained after five consecutive adsorption–desorption cycles.

## Figures and Tables

**Table 1:** Kinetic parameters for Pb(II) adsorption on the CNC-g-P(AA/AM)-0.5, CNC-g-P(AA/AM)-1, CNC-g-P(AA/AM)-2, and CNC aerogels.

Sample	Q_e,exp_ (mg g^−1^)	Pseudo-First-Order			Pseudo-Second-Order		
		k_1_ × 10^−3^ (min^−1^)	q_e1,cal_ (mg g^−1^)	R^2^	k_2_ × 10^−4^ (g mg^−1^ min^−1^)	q_e2,cal_ (mg g^−1^)	R^2^
CNC aerogel	169.6	7.60	52.16	0.906	6.19	173.0	0.992
CNC-g-P(AA/AM)-0.5	217.3	7.34	157.4	0.933	1.10	220.3	0.995
CNC-g-P(AA/AM)-1	223.8	8.94	176.5	0.975	1.51	225.7	0.998
CNC-g-P(AA/AM)-2	210.8	6.12	143.8	0.924	1.35	207.5	0.999

**Table 2:** Kinetic parameters of the intra-particle diffusion model.

	Parameters	CNC-g-P(AA/AM)-0.5	CNC-g-P(AA/AM)-1	CNC-g-P(AA/AM)-2	CNC Aerogel
Stage 1	R^2^	0.985	0.978	0.999	0.981
	k_i,1_ (mg g^−1^ min^1/2^)	20.35	19.53	19.31	23.72
	C (mg g^−1^)	−25.65	−10.46	−22.69	3.831
Stage 2	R^2^	0.972	0.928	0.991	0.914
	k_i,2_ (mg g^−1^ min^1/2^)	0.338	0.194	0.372	0.718
	C (mg g^−1^)	183.81	192.96	176.14	135.18

**Table 3:** Adsorption isotherm parameters for the Langmuir and Freundlich models.

Sample	Langmuir Model			Freundlich Model		
	$Q_{m}\;(\mathrm{mg}\;g{-}^{1})$	*b*	R^2^	*n*	$k_{f}$	R^2^
CNC aerogel	208.3	0.046	0.998	2.19	19.6	0.934
CNC-g-P(AA/AM)-0.5	326.8	0.026	0.998	1.95	22.5	0.933
CNC-g-P(AA/AM)-1	366.3	0.060	0.998	2.19	33.3	0.933
CNC-g-P(AA/AM)-2	302.1	0.024	0.998	2.20	27.0	0.939

## Data Availability

The raw/processed data required to reproduce these findings can be shared via contacting to the corresponding author e-mail.
